# Pharmacologic reductions of total tau levels; implications for the role of microtubule dynamics in regulating tau expression

**DOI:** 10.1186/1750-1326-1-6

**Published:** 2006-07-26

**Authors:** Chad A Dickey, Peter Ash, Natalia Klosak, Wing C Lee, Leonard Petrucelli, Michael Hutton, Christopher B Eckman

**Affiliations:** 1 Department of Neuroscience, Mayo Clinic Jacksonville, 4500 San Pablo Rd., Jacksonville, Florida, 32224, USA

## Abstract

The microtubule-associated protein tau (MAPT) is a pathological component of several neurodegenerative diseases and clinical dementias. Here, we have investigated the effects of a series of commercially available FDA-approved compounds and natural products on total tau protein levels using a cell-based approach that allows for the rapid and efficient measurement of changes in protein expression.

**Results:**

The compounds that reduced tau largely fell within 3 functional categories with the largest percentage being microtubule regulators. Several of these candidates were validated in both a human neuroglioma and a human neuroblastoma cell line. While these drugs lead to a rapid reduction in tau protein levels, a selective decrease in MAPT mRNA expression was also observed.

**Conclusion:**

These findings suggest that the identified compounds that reduce tau levels may act either through direct effects on the MAPT promoter itself or by altering a feedback transcriptional mechanism regulating MAPT transcription. This is particularly interesting in light of recent evidence suggesting that MAPT 5' UTR mutations in late-onset PD and PSP cases alter the expression of tau mRNA. In fact, one of the compounds we identified, rotenone, has been used extensively to model PD in rodents. These observations may provide key insights into the mechanism of tau turnover within the neuron while also providing the first evidence that selectively reducing tau protein levels may be possible using compounds that are FDA-approved for other uses.

## Background

The microtubule-associated protein tau (MAPT) has been demonstrated to be an integral component of axon formation and neuronal transport, and serves as a substrate for multiple cellular signaling pathways [1,2]. However, in several neurological disorders with clinical dementia, tau apparently loses its normal function and begins to aberrantly accumulate into various types of pathological inclusions. Mutations within the *MAPT *gene [3,4], were shown to result in the formation of neurofibrillary tau aggregates leading to FTDP-17 (frontotemporal dementia with parkinsonism linked to chromosome 17), thus providing conclusive evidence that abnormalities in tau are sufficient to produce neurodegeneration and dementia. While Alzheimer's disease (AD) is indeed the predominant dementia demonstrating consistent tau pathology, the accumulation of abnormally phosphorylated tau species is also a primary histopathologic hallmark of several other heritable dementias, including corticobasal degeneration (CBD), parasupranuclear palsy (PSP) and frontotemporal dementia (FTD). In addition, post-mortem assessments of many late onset Parkinson's disease (PD) cases demonstrate abnormal tau aggregation, further suggesting the central role that this protein may have in a host of neurological disorders not associated with amyloid deposition as with AD.

Recent evidence has indicated that the abnormal intracellular accumulation of tau may ultimately be self-perpetuating [5], and this conversion of tau has been proposed to be a toxic gain-of-function phenomenon [6]. The recent generation of viable and seemingly normal MAPT ^-/- ^mice has raised the possibility that neurons may be tolerant of perturbations in the overall levels of tau protein. Collectively, these data suggest that reductions in tau may be a viable therapeutic application. In further support of this notion, Santacruz et al. also recently demonstrated that reducing the expression of MAPT mRNA in a Tet-Off inducible tau mouse model reversed the cognitive deficits and neuronal loss in these mice without reducing the number of tau aggregates [7]. Therefore, tau itself rather than the aggregates and tangles, may serve as a valid therapeutic candidate, particularly in cases caused by familial mutations of the MAPT locus. These studies demonstrate the need for compounds to facilitate the removal of either the total pool of tau or specific aberrant forms of tau (i.e. hyperphosphorylated, misfolded, etc.). Unfortunately, however, the feasibility of large scale efforts to identify modifiers of endogenous protein species has proven technically challenging. We recently developed a novel technique to quantitatively measure various tau species, including an assay to analyze total tau protein while simultaneously measuring GAPDH levels as an estimate of cell viability [8]. Using this platform, we have screened a library of off-patent compounds and have identified several that are capable of reducing tau levels in brain-derived cell lines.

## Results

**Figure 1:**
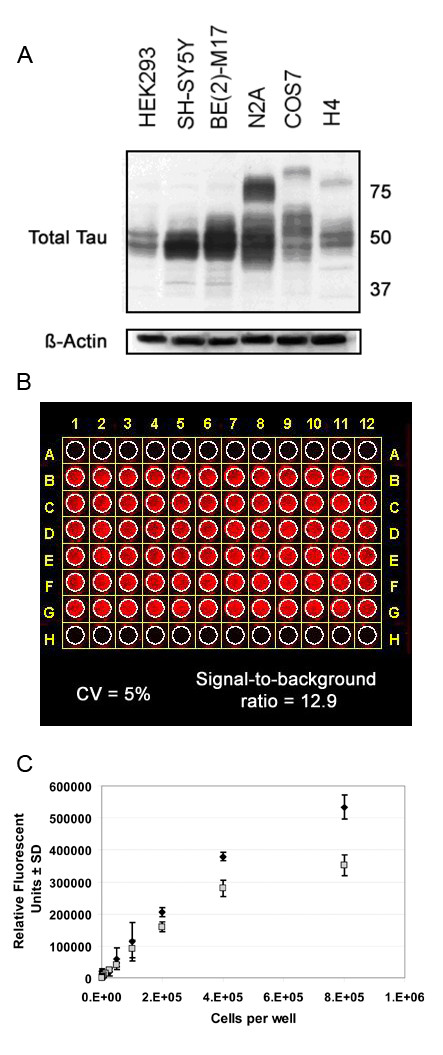
**In-Cell Western Optimization**. (A) Tau levels from multiple cell lines were assessed by Western blot. H4 neuroglioma cells were chosen for the assay for several reasons including relatively robust tau expression, adhesion properties and human neural lineage. β-actin levels were assayed to control for variations in protein loading. (B) H4 cells were plated in a 96-well plate and tau immunoreactivity was determined by fluorescent (680 nm) secondary antibody detection using the *Odyssey *infrared scanner. Conditions were optimized to produce a low 5% well-to-well variation across the plate represented as CV (coefficient of variance) for rows B-G, and a signal-to-background ratio of >10-fold (12.9; background is empty wells in rows A and H). (C) Cells were plated at the densities indicated on the x-axis. Relative Fluorescence for triplicate wells were averaged and the standard deviation was calculated. Black diamonds (◆) indicate tau immunoreactivity with detection at 680 nm; gray boxes (■) indicate GAPDH immunoreactivity with detection at 800 nm

A primary pathological contributor to several dementias is the abnormal accumulation of the tau protein. Therefore reducing the overall levels of this protein may hold therapeutic relevance. We have developed a quantitative cell-based screening methodology for measuring intracellular tau protein levels to identify off-patent and FDA-approved compounds that may hold efficacy against this major protein component of AD pathology. Preliminary Western blot analyses revealed that, combined with their robust adhesiveness, H4 human neuroglioma-like cells have substantial tau immunoreactivity corresponding to both 3 and 4 repeat human tau isoforms (Fig 1A). Therefore, we chose this line for all subsequent screens to assess total tau levels. To determine the dynamic linear range for tau and GAPDH in the H4 cells using the In Cell Western assay, cells were plated at different seeding densities and incubated for 48 hours. We determined that for tau detection with the 680 nm fluorescent-labeled anti-rabbit secondary antibody and GAPDH detection with the 800 nm fluorescent-labeled anti-mouse secondary antibody, the optimal range for detecting tau was at a point when H4 cells were seeded at a density between ~3 × 10^4 ^and ~4 × 10^5 ^per well (Fig 1C). We then determined that the coefficient of variance was 5% for total tau immunoreactivity and the signal-to-background ratio was approximately 12.9×. We therefore seeded 8 × 10^4 ^cells/well for all subsequent investigations.

Using this ICW paradigm, we screened a commercially available chemical library containing ~880 functionally diverse molecules (85% of which are commercially marketed) for compounds that reduced total tau levels. Initial analyses revealed that 26 compounds (3 %) were shown to significantly (p < 0.05) reduce tau immunoreactivity (IR) relative to GAPDH IR (Table 1). Of these 26 compounds, 9 resulted in >25% reduction in GAPDH IR (Table 1; italics). Two compounds, epicatechin and betamethasone, were falsely identified by our initial analyses as they increased GAPDH IR without changing tau IR (Table 1; small caps) and this altered the tau/GAPDH ratio used to initially identify compounds. Nine compounds caused a >25% reduction in tau levels with <10% reductions in GAPDH IR, perhaps representing the most optimal category for therapeutic relevance (Table 1; bold). The remaining six compounds did reduce tau by at least 19% (ciclopirox) or more, with differing effects on GAPDH levels (Table 1; bold-italics).

**Table 1:** Tau-reducing compounds identified from initial in-cell western screening assay

***Functional Grouping***	***Drug Name***	***Tau IR as a % Of Vehicle Control***	***GAPDH IR as a % Of Vehicle Control***
**Aggregation inhibitors**	*Daunorubicin*	*21 ± 0*	*52 ± 6*
	**Diazaquone**	**66 ± 3**	**106 ± 6**
	**Methylene Blue**	**72 ± 6**	**112 ± 6**
	EPICATECHIN	100 ± 4	140 ± 5
	*Doxorubicin HCl*	*41 ± 3*	*56 ± 6*

**Antibiotics**	**Alexidine HCl**	**57 ± 0**	**91 ± 9**
	***Gramicidin***	***58 ± 0***	***84 ± 0***
	***Ciclopirox ***	***81 ± 2***	***109 ± 6***

**Anti-proliferatives**	*Camptothecine*	*38 ± 0*	*71 ± 1*
	*Azacytidine-5*	*40 ± 0*	*56 ± 3*
	*Emetine diHCl*	*51 ± 11*	*69 ± 2*
	***Mycophenolic Acid***	***77 ± 0***	***100 ± 7***
	***Azaguanine-8***	***79 ± 0***	***88 ± 3***

**Microtubule regulators**	*Paclitaxel*	*47 ± 2*	*61 ± 4*
	**Colchicine**	**44 ± 0**	**93 ± 6**
	**Albendazole**	**44 ± 4**	**93 ± 12**
	*Podophyllotoxin*	*37 ± 0*	*63 ± 2*
	*Nocodazole*	*38 ± 0*	*61 ± 3*
	*Mebendazole*	*45 ± 3*	*69 ± 4*
	***Fenbendazole***	***51 ± 2***	***76 ± 9***
	**Chelidonine **	**67 ± 0**	**96 ± 4**
	**Rotenone**	**72 ± 0**	**99 ± 1**

**Receptor Antagonists**	***Lanotoside C***	***61 ± 2***	***79 ± 2***
	***Lasalocid Na salt***	***77 ± 2***	***96 ± 2***

**Steroids**	BETAMETHASONE	99 ± 0	146 ± 13
	**Norethindrone**	**69 ± 1 **	**110 ± 3**

*Italics *= Drugs that reduced tau levels, but that reduced GAPDH levels by >25% indicating some toxicity; SMALL CAPS = Drugs that increased GAPDH levels without reducing tau levels; **Bold **= Drugs resulting in >25% reductions in tau levels with <10% reductions in GAPDH levels; ***Bold Italics ***= Drugs demonstrating <25% reductions in tau levels and/or 10–25% changes in GAPDH levels. IR indicates immunoreactivity. SD indicates standard deviation. Compounds are grouped based on their functional class.

**Figure 2:**
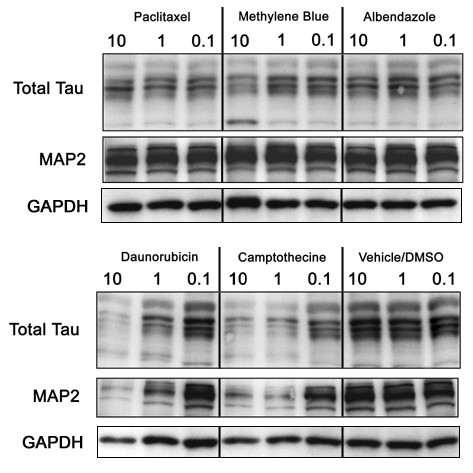
**Validation of candidate compound efficacy for tau reductions in H4 neuroglioma cells by standard Western blot**. H4 neuroglioma cells were treated at 90% confluency for 24 hours with respective μM concentrations of drugs shown at the top of each lane. Cells were then harvested and homogenized, normalized for protein concentration and subjected to SDS-PAGE followed by probing for total tau, MAP2 and GAPDH. We validated the efficacy of 5 compounds by Western blotting demonstrating that two of these compounds, daunorubicin and camptothecine, show capacity to reduce both MAPs and GAPDH compared to vehicle (bottom right), while the other 3 compounds, paclitaxel, methylene blue and albendazole, all appear to have specific effects on tau levels compared to vehicle.

**Figure 3:**
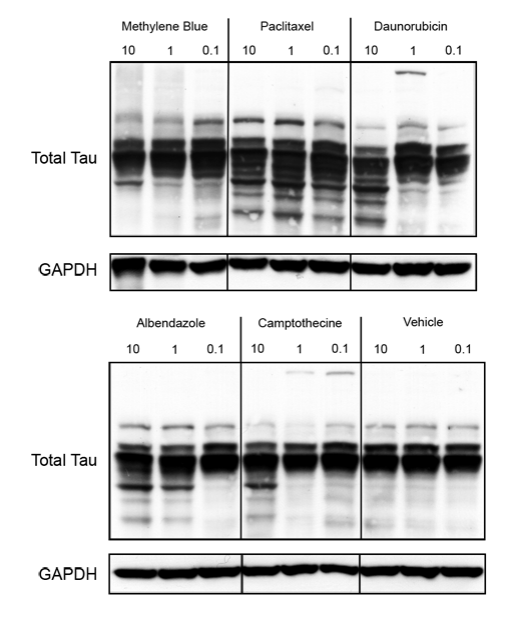
**Validation of candidate compound efficacy for tau reductions in M17 neuroblastoma cells by standard Western blot**. M17 neuroblastoma cells were treated at 90% confluency for 24 hours with respective μM concentrations of drugs shown at the top of each lane. Cells were then harvested and homogenized, normalized for protein concentration and subjected to SDS-PAGE followed by probing for total tau and GAPDH. All 5 compounds altered tau levels. Several compounds caused fragmentation of tau relative to vehicle treated cells. Note the high molecular weight species present at the 1 μM dose of daunorubicin and the 1 and 0.1 μM doses of camptothecine, perhaps indicating the formation of a multimeric structure during tau degradation.

To further identify optimal therapeutic candidates, we performed a dose response on the H4 cells with the ICW methodology (100 μM to 10 nM) along with LDH analyses. Thirteen compounds were chosen from the initial 25 based on several factors including the potency of the tau IR reductions from the primary screens, relevance from literature and commercial availability. Only 6 compounds demonstrated a therapeutic window where the concentration that reduced tau levels by 50% was 10-fold less than the concentration that was toxic to 50% of the cells (as assessed by LDH levels). Five of these compounds (daunorubicin, methylene blue, camptothecine, paclitaxel and albendazole; betamethasone was excluded due to increased GAPDH IR in primary screens), were further characterized for their effects on tau by Western blot and qRT-PCR analyses. Western blot analysis revealed that tau levels were significantly reduced in a dose-dependent manner for all 5 compounds relative to vehicle treated cells (Figure 2). Daunorubicin and camptothecine demonstrated similar reductions in MAP2 levels and slight reductions in GAPDH IR whereas paclitaxel, methylene blue and albendazole appear to reduce tau specifically (Figure 2). To determine the universality of these effects, we then assessed efficacy in both the M17 and N2A cell lines. Interestingly, only tau within the human M17 neuroblastoma cell line (Figure 3) was responsive to treatment with the 5 lead candidates, while N2A tau levels remained largely unchanged (not shown). Thus, these effects were not replicated in a murine neuroblastoma cell line, suggesting that the compounds identified may not have sufficient efficacy in mouse model systems, despite efficacy in two different human CNS derived cell lines.

**Figure 4:**
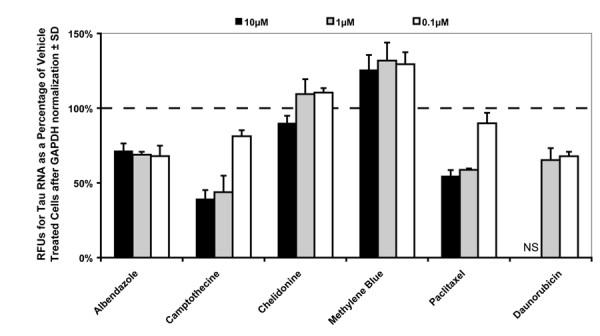
**Compounds that reduce tau protein levels also affect mRNA expression**. H4 cells were treated in triplicate with 10, 1, and 0.1 μM of compounds listed on x-axis. Quantitative real time PCR was used to determine expression levels of tau relative to GAPDH. Relative fluorescent units (RFUs) were determined and presented here as a percentage of the vehicle-treated controls. "NS" indicates no RFUs detected.

Real time PCR was then used to determine if tau mRNA expression in the H4 cell line was altered following treatment with these 5 candidates. Interestingly, daunorubicin, paclitaxel, albendazole and camptothecine all reduced tau mRNA levels relative to GAPDH (Fig 4). Camptothecine and paclitaxel caused dose responsive reductions in tau mRNA whereas albendazole and daunorubicin similarly reduced tau levels at each concentration (with the exception of the 10 μM daunorubicin concentration that had no detectable mRNA expression for either tau or GAPDH). Conversely, methylene blue increased tau mRNA expression relative to GAPDH despite reducing tau protein levels (Fig 4). Chelidonine was included as a control compound that reduced tau levels by primary screens but did not change mRNA expression.

## Discussion

While most recent AD therapeutic development has focused on the removal of Aβ or prevention of its aggregation into plaques, the recent findings by Oddo et al and Santacruz et al. that tau accumulation can only be reversed at an early stage in its AD pathogenesis highlights the need for therapeutic strategies to slow or even stop tau accumulation in this disease and other hereditary tauopathies [5,7]. Several strategies have recently been suggested that target various pathologically associated tau species, including hyperphosphorylated and aggregation-competent species (For review see [6]).

While the tau protein was originally characterized as a late-stage component of AD pathology, the identification of mutations within the MAPT gene itself in patients with clinical dementia and a parkinsonian phenotype (i.e. FTDP-17) [3] demonstrated that tau, in and of itself, is capable of causing neurodegenerative disease. In fact, several studies have now demonstrated that genetic variation in the critical 5' and promoter region of the MAPT locus was associated with typical late-onset PD (LOPD) [12,13] and irregular tau accumulation has been observed in both parkinsonism kindreds and sporadic LOPD cases [14]. Furthermore, a recent report demonstrated that tau transcriptional activity was impaired by introduction of a single nucleotide polymorphism found on the H1 haplotype of progressive supranuclear palsy patients [15]. Thus, altered expression of the tau protein may indeed contribute to the onset of parkinsonism, including LOPD. While both α-synuclein and tau accumulation are often a neuropathological feature of clinically diagnosed PD patients following post-mortem examination, a recent study demonstrated that adenoviral over-expression of tau, but not α-synuclein, was sufficient to promote dopaminergic neuronal loss and impair rotational behavior in otherwise normal rats, further supporting an important role for tau in the pathogenesis of PD [16].

Here, based on findings that mice entirely deficient of tau expression are quite viable [17,18] and the notion that generally reducing the pool of tau available for further aggregation may hold therapeutic potential after disease onset, we analyzed a small library of predominantly marketed compounds for their ability to reduce total tau protein levels. Using an ICW assay [8,9], we have demonstrated that several compounds indeed appear able to reduce tau protein levels, and that out of 25 compounds identified, 9 were involved with microtubule dynamics and 4 were putative protein filament/aggregation inhibitors. We demonstrated that several of these drugs were able to selectively lower tau levels without affecting another microtubule-associated protein, MAP2. In addition, several (5) anti-proliferative compounds were identified from our screens, further emphasizing the role that tau plays in cell division and the potential sensitivity of its expression based on the functional requirements of the cell at any given time.

We classified tau-reducing compounds based on their functional capacity as cited in the current body of literature. Inasmuch, a recent report by Pickhardt and colleagues demonstrated that anthraquinones were able to dissolve paired helical filaments of tau and inhibit its aggregation [20]. Earlier work has also demonstrated that doxorubicin regulates both tau metabolism [21] and disrupts microtubules [22]. Therefore, our identification of anthraquinones, such as doxorubicin and daunorubicin, as reducers of total tau levels, is not only consistent with previous findings, it also provides additional support for the idea that tau levels may be highly dynamic based on the requirement of the cell for the generation of microtubule networks. Perhaps more importantly, these findings suggest that the degradation of tau is a highly ordered process that requires precise synchronicity at both the translational and transcriptional levels due to the multiple cleavage fragments, the reductions in MAPT mRNA levels, and even the appearance of higher molecular weight tau species that are produced by these compounds. We also noticed that while toxicity was certainly observed with several compounds, tau levels were significantly lower compared with GAPDH levels. This was somewhat surprising as the half-life of both tau protein and mRNA are thought to be relatively long-lived [23,24] compared to other molecules. Certainly, while under normal conditions tau might be quite stable, our results suggest that compounds targeting the tubulin, the substrate of tau, promote the rapid and ordered removal of tau from the cell. This may indicate that impaired degradation of the tau protein could ultimately facilitate its abnormal accumulation in a diseased condition such as AD and PD.

Follow-up studies in the H4 cell line revealed that a primary mechanism by which these compounds elicited tau reductions was likely due to reduced transcriptional activity (Fig 4). Some conclusions regarding specificity of this effect can be drawn as it has previously been demonstrated that tau mRNA is an extremely stable transcript in neurons due to 3' UTR structural motifs [24]. These results perhaps indicate that the MAPT 5' UTR is being directly modulated by these compounds, or that microtubule destabilization activates a negative feedback pathway that reduces tau expression. It is therefore possible that one of the fragments produced by treatment with these compounds (Fig 3) is also negatively regulating MAPT mRNA expression. This could link the genetic association found in the 5' MAPT locus for LOPD that seems to lead to enhanced tau expression [Kwok] [Skipper] with a functional consequence of impaired intraneuronal transport due to microtubule breakdown and altered tau expression (i.e. presynaptic accumulation of α-synuclein and mitochondrial breakdown, two classical features of PD). This finding also supports earlier work by Litman et al., demonstrating that tau mRNA itself co-localizes with intact microtubules within axons [25], and therefore, disruption of microtubules may lead to local reductions in both tau protein and mRNA levels. This could also suggest that MAPT mRNA is perhaps locally translated at the pre-synapse, providing for a mechanism of rapid regulation that is not impeded by classical protein trafficking from the nucleus. Therefore, the alteration of microtubule stability may in fact lead to altered expression levels of tau.

When human neuroblastoma cells were treated with several of these same compounds and subjected to Western analysis, marked degradation of tau was observed and in some cases a high molecular weight (HMW) tau species emerged. Earlier studies identified this HMW tau as a 110 kDa species produced from an alternatively spliced transcript of tau that is predominantly found in the peripheral nervous system [26,27]; however the functional capacity of HMW tau remains unclear. Another interesting finding was the lack of detectable tau reductions in the mouse N2A neuroblastoma cell line following treatment with these same compounds. Human and mouse tau are processed quite distinctly, in that adult mice exclusively produce exon 10 (+) tau isoforms while normal adult humans produce both exon 10 (+) and exon 10 (-) tau isoforms at a 1:1 ratio [28]. In addition, despite robust Aβ deposition in mice transgenic for mutant forms of the amyloid precursor protein (APP), neither tau accumulation nor tangle formation has been observed in these animals. Thus, mouse tau is distinctly different from human tau and therefore it is not surprising that compounds affecting human tau are not capable of altering mouse tau. It also suggests that *in vivo *validation of these compounds will likely require the use of mice transgenic for human tau or even mice that have been humanized for the MAPT gene [29].

## Conclusion

Certainly, while all of the compounds identified have unique properties and functions, our results indicate that microtubule disruption is a primary modifier of tau expression and levels. While these compounds have typically been used to arrest cell division, it has yet to be determined what their impact might be in terminally differentiated neurons that utilize microtubules and their associated proteins in an entirely different manner from the rest of the body. The recent study by Zhang et al. demonstrated that *in vivo *administration of the microtubule stabilizer *Paxceed *prevented tau sequestration and improved axonal transport in a mouse model of tauopathy, providing the first indication that compounds affecting microtubule integrity might have therapeutic potential for neurodegenerative diseases involving tau [30]. Unfortunately tau levels were not assessed in the mice transgenic for human tau in this study; however, based on our findings here it would be predicted that tau levels were reduced in those mice. Thus, in patients already suffering from heritable dementia associated with mutations within the MAPT gene, perhaps reducing the available pool of the pathogenic substrate could hold immediate clinical promise, particularly as many of the compounds identified in this analysis are FDA-approved and currently being used by physicians to treat other various disorders.

## Materials and methods

### Cell culture and drug treatments

The human H4 neuroglioma, human BE(2)-M17 neuroblastoma (M17) and mouse Neuro2a (N2A) neuroblastoma cell lines were maintained in T-75 flasks with Opti-MEM (Invitrogen) with 10% FBS and antibiotic. N2A cells were supplemented with 2 mM glutamine. New cultures were started monthly and passaged thereafter twice weekly with trypsin. In preparation for drug treatment and In-Cell Western analysis, H4 cells were passaged into 96-well clear bottom plates (BD Falcon) at a density of 8 × 10^4 ^cells/well and incubated overnight. Cells were treated in triplicate for 24 hours with a single concentration (normally 1–10 μM) of each compound from the Prestwick (880 compounds) library or vehicle (DMSO). Based on our previous studies, EC82 (Conforma Therapeutics) [9] was added to each plate for use as a positive control for tau reductions. For dose response studies, 100, 10, 1, 0.1 and 0.01 μM concentrations of drug were added to wells in duplicate for 24 hours. For preparation of homogenates for normal Western blot analyses, cells were passaged into 6-well plates, incubated overnight and treated the next day with drug or vehicle at indicated concentrations. Cells were then incubated for 24 hours and harvested in lysis buffer containing (50 mM Tris-HCl pH 7.4, 1 M NaCL, 0.1% Triton-X, 5 mM EDTA) plus 1% SDS, PMSF, and both a protease and phosphatase inhibitor cocktail.

#### Western blot analysis

Cell lysates were sonicated and protein concentrations were measured by a standard BCA assay (Pierce). The samples were then heated in Laemmli's buffer and equal amounts of protein were loaded into 12-well 10% Tris-HCl gels (Bio-Rad). Protein expression was then analyzed by Western blot using anti-human tau (1:2000; DAKO), anti-MAP2 (1:1000; Cell Signaling) and anti-GAPDH (1:2000; BioDesign) antibodies. HRP-conjugated secondary antibodies were applied and visualized by ECL treatment and exposure to film.

#### In-Cell Western blotting and quantitation

In-Cell Western analysis was performed as previously described [8,9]. Briefly, 50 μl of media from the 96-well plates were removed for LDH analysis to detect toxicity (Cytotox 96 Assay Kit, Promega) and the remaining media was aspirated. Cells were fixed with 3.7% formaldehyde in PBS for 20 minutes and then permeabilized with three 10 minute washes in PBS + 0.1% Triton-X 100 (BioTek Instruments Inc.). A proprietary lipid-based blocking buffer (LBB; Li-Cor) was added for two hours, followed by overnight incubation with rabbit anti-human tau (1:500; DAKO) and mouse anti-human glyceraldehyde-3-phosphate dehydrogenase (GAPDH; 1:1500; BioDesign) antibodies in a 1:1 buffer of LBB and PBS + 0.2% Tween20. After three 10 minute washes in PBS + 0.1% Tween-20, secondary detection was carried out using two infrared fluorescent dye conjugated antibodies in 1:1 buffer of LBB and PBS + 0.4% Tween20; one absorbing at 680 nm (AlexaFluor 680; Molecular Probes) and the other absorbing at 800 nm (IRDye 800 CW; Rockland). After an hour incubation and washing, the targets were simultaneously visualized using the Odyssey Infrared Imaging Scanner (Li-Cor, Lincoln, NB). Relative fluorescent units for drug-treated samples were divided by vehicle controls to determine percent change in expression of both tau and GAPDH levels relative to control. The student t-test was used to determine significant toxicity effects due to drug treatment based on the expression levels of GAPDH, and then a separate student t-test was used to determine significant differences between tau and GAPDH levels in treated cells.

#### qRT-PCR

RNA was isolated from cells and analyzed by qRT-PCR as previously described [10,11]. Briefly, Total RNA from H4 cells treated at 3 different concentrations (10 mg/ml, 1 mg/ml and 0.1 mg/ml) in duplicate was isolated using the RNeasy 96 technology from Qiagen and reverse transcribed with MMLV reverse transcriptase and 1 M Betaine. A standard curve was established within the reverse transcription reaction by adding total RNA (template) from untreated H4 cells covering 3 logs. 5 ng of total RNA from all samples were added to individual wells within the reverse transcription reaction for comparison to the standard curve. Primers for SYBR green qPCR analysis of 28S rRNA levels and both GAPDH and Total Tau mRNA levels were generated as previously described [10]. Experimental wells containing 25 μl PCR reactions were run in 384-well plates. PCR was run on the ABI 7900 as follows; 1 cycle of 95°C for 15 minutes followed by 40 cycles of 95°C for 15 seconds and 60–65°C for 1 minute. None of the primer pairs demonstrated more than 1 peak of fluorescence from melt curve analysis. The standard curve was calculated by plotting the threshold cycle (Ct) against the log nanogram quantity of RNA added to the RT reactions. A linear regression was performed and the slope, relating Ct to log ng RNA, was calculated, and converted to a mass quantity of standard RNA. These mass values for the genes of interest were then normalized to 28S ribosomal RNA mass values and then divided by GAPDH mRNA to determine fold-change in mRNA expression relative to the standard RNA pool. These fold change values for samples in the vehicle treated vs. drug treated were analyzed for significance using Student t-test.

## Authors' contributions

CD designed, performed and analyzed all data generated from ICW assays, qPCR and Western blotting. He composed the paper and interpreted the findings. PA and NK assisted with cell culture and Western blot analyses. WL and LP provided interpretation of the results and intellectual conception. MH and CE provided initial conception of the program, secured funding for the research, developed the direction of the project, and contributed to the writing of the manuscript.

**Table 2:** Therapeutic window for most favorable candidate compounds

	***TC50 (μM)***	***IC (μM)***
**Paclitaxel**	**0.1**	**0.01**
**Camptothecine**	**0.1**	**0.01**
*Fenbendazole*	*10*	*10*
*Mebendazole*	*1*	*1*
**Albendazole**	**10**	**1**
*Norethindrone*	*100*	*100*
**Betamethasone**	**>100**	**100**
**Methylene Blue**	**>100**	**10**
*Rotenone*	*1*	*1*
**Daunorubicin**	**1**	**0.1**
*Chelidonine*	*10*	*10*
*Alexidine*	*10*	*10*
*Podophyllotoxin*	*0.1*	*0.1*

The concentration that was toxic to 50% of the cells (TC50) and the concentration that significantly reduced tau levels (IC) was determined for several compounds identified from screening
